# Computed tomography derived bone density measurement in the diabetic foot

**DOI:** 10.1186/s13047-017-0192-7

**Published:** 2017-03-03

**Authors:** Alex Barwick, John Tessier, James Mirow, Xanne Janse de Jonge, Vivienne Chuter

**Affiliations:** 1 University of Newcastle, 10 Chittaway Rd, Ourimbah, NSW 2258 Australia; 2 University of Newcastle, University Dr, Callaghan, NSW 2308 Australia; 3 Hunter Imaging Group, 48 Thomas Street, Cardiff, NSW 2285 Australia

**Keywords:** Bone, Diabetes, Foot, Reliability

## Abstract

**Background:**

The accurate and reliable measurement of foot bone density is challenging and there is currently no gold standard technique. Such measurement is particularly valuable in populations at risk of foot bone pathology such as in those with long term diabetes. With research and development, computed tomography may prove to be a useful tool for this assessment. The aim of this study was to establish the reliability of a novel method of foot bone density measurement in people with diabetes using computed tomography.

**Methods:**

Ten feet in people with diabetes were scanned with computed tomography twice with repositioning. Bone density (in Hounsfield units) was assessed in the trabecular and cortical bone in all tarsals and metatarsals. Reliability was assessed with intra-class correlation coefficients (95% confidence intervals), limits of agreement and standard error of measurement.

**Results:**

The reliability of the trabecular density of most bones was excellent with intra-class correlation coefficients ranging from 0.68 to 0.91. Additionally, cortical bone density showed fair to good reliability at the talus (0.52), calcaneus (0.59), navicular (0.70), cuboid (0.69), intermediate cuneiform (0.46) and first metatarsal (0.61).

**Conclusions:**

The study established the reliability of a practical method of assessing the trabecular and cortical foot bone density using computed tomography scanning. This methodology may be useful in the investigation of foot bone disease occurring in diabetes and its early diagnosis, intervention and assessment of treatment efficacy. Further development of this method is warranted.

**Electronic supplementary material:**

The online version of this article (doi:10.1186/s13047-017-0192-7) contains supplementary material, which is available to authorized users.

## Background

People with diabetes have an increased risk of bone fracture both centrally and peripherally [1, 2]. In the presence of diabetes, bone regulation is disturbed by hypercalcuria, an increase in reactive oxygen species, increased polyol pathway activity and the non-enzymatic glycosylation of bone [3]. Accordingly, diabetes has been shown to affect bone mineral density (BMD) and its microstructure [4], reduce fracture resistance, and impair bone regeneration [5].

These generalised changes to bone regulation, in addition to presence of peripheral diabetes complications, are proposed to leave individuals susceptible to Charcot neuroarthropathy [6]. This is a disease characterised by extreme alterations to bone throughout its natural history including rapid bone resorption and resulting reduction in density followed by new bone formation, increases in density and joint fusions [6]. Improvements in foot imaging techniques are useful for the investigation of such disease processes and may assist in early diagnosis and monitoring of treatment effectiveness [7].

Traditional techniques of assessing BMD lack the accuracy to establish density in foot bones due to the intricate nature of foot bone morphology [8]. For example, dual-energy x-ray absorptiometry is widely used in the assessment of bone density centrally, but does not have the capability to distinguish between the small bones within the foot that sit closely together [8]. Similarly, ultrasound has been used to assess the integrity of the calcaneus, but is impractical to use on the rest of the bones of the foot, which are also prone to fracture and Charcot neuroarthropathy in people with diabetes [9]. The technical capabilities of computed tomography (CT) may be able to overcome these difficulties [10]. Computed tomography is a non-projection technique that can not only distinguish between individual bones in the foot, but also between trabecular and cortical bone [11]. This is useful as trabecular bone is more metabolically active and therefore may be more affected by disease processes [11]. If proven to provide accurate and reliable, CT may provide important information on foot bone integrity.

Computed tomography can be used to assess BMD through segmentation, as well as through the analysis of single slices. Peripheral quantitative computed tomography (pQCT) provides volumetric analysis of individual bone slices, but is complicated by the need to replicate scan location based on bony landmarks. As such, it has been restricted to the radius and tibia [11], though it can also reliably be used to assess the second metatarsal [12].

Three-dimensional segmentation of the tarsals and metatarsals has demonstrated excellent precision [8], although the bone registration and segmentation process is very time consuming and requires specialised software. This may be avoided by averaging several slices from the obtained three-dimensional images, providing a simple, but sufficient, means of assessing foot bone quality in at-risk populations on a larger scale. To the authors’ knowledge there has been no assessment of the reliability of bone density analysis of single slices of foot bones obtained from three-dimensional acquisition techniques.

The aim of this study was to investigate the reliability of a novel method of assessment of cortical and trabecular BMD of the tarsals and metatarsals of the feet in those with diabetes using CT.

## Methods

Ten older people with type 2 diabetes were recruited from a podiatry clinic in New South Wales, Australia to participate in this test-retest design method reliability study. Participants were excluded if they were pregnant, took corticosteroids, or hormone replacement therapy, had osteoporosis (all participants underwent Dual x-ray absorptiometry), chronic renal failure, current bilateral foot ulceration, Charcot neuroarthropathy, malignancy, endocrine disorders (other than diabetes), a recent history of foot trauma (in both feet) or had participated in research involving ionising radiation in the previous 12 months. Demographic characteristics of the participants are shown in Table 1. Participants were taking either insulin (1), oral hypoglycaemic agents (3), both (1) or were diet controlled (4). No participant was taking hypoglycaemic agents associated with risk of fracture. The study was approved by the University of Newcastle Human Research Ethics Committee and informed consent was obtained from all participants.

**Table 1:** Participant demographic information

Male/Female	8/2
Age (SD)	72.90 (4.56)
BMI (SD)	31.30 (5.01)
Diabetes duration (SD)	12.15 (11.81)

*BMI* body mass index

SD - standard deviation

An Aquilion One 320 slice CT scanner (Toshiba Medical Systems, Japan) was used for all examinations. One radiographer performed all aspects of the examinations, including participant positioning, scanning and acquisition of measurements. A pre-planned program was utilized for each examination. No adjustments to the pre-planned program were made for any participant.

Volume acquisition was utilised with the following settings applied: CTDIvol 7.2 mGy; dose-length product 115.9 (mGy x cm); 120 kV; 150 mA; rotation time 0.5 s; range 16 cm; display field of view medium or large (depending on foot size). The participant’s right foot was scanned twice, except where prohibited by injury or amputation, in which case the left foot was scanned (three cases). Each participant was placed in a recumbent position on the CT table, offset to the participants’ contralateral side in order to allow a more midline position for the lower extremity that was to be scanned. The contralateral knee was flexed to prevent scanning of this foot. The degree of angulation of the contralateral leg was determined by the comfort of the patient to assist in maintaining the desired position throughout examination in an effort to prevent any movement artefact and the need for repeat scanning.

The foot to be scanned was placed against a wooden box with the ankle in a neutral position as close to 90° to the table surface as possible. The foot was scanned using the pre-planned program and resultant images were assessed by the radiographer for any movement artefact and to ensure that all anatomical areas were covered. Once the radiographer ratified the imaging data, the participant was removed from the CT table. This whole process was performed twice for each individual participant.

In all ten feet, all seven tarsals and the five metatarsals were assessed in the axial plane of reconstruction. All images were viewed with a window level of 350 and a window width of 2700. Images were reconstructed 0.5 mm thick at intervals of 0.25 mm. Three random slices were obtained from the body of each of the 12 bones. The radiographer selected appropriate regions from the slices of each participant and Hounsfield unit (HU) measurements were obtained. The largest region of interest possible was traced in the trabecular bone and three regions of interest were taken from the cortical bone from each slice. These were averaged.

Statistical analysis was performed in SPSS Version 22 for Windows (SPSS Inc, Chicago, USA). Test-retest reliability between scans 1 and 2 was determined with intra-class correlation coefficients (ICC) and 95% confidence intervals (CI) for all twelve bones for both cortical and trabecular bone. A mixed 2 way (3, 1) model was used. Interpretation of ICC values was in accordance with Fleiss [13]: > 0.75 considered excellent reliability, 0.40 to 0.75 considered fair to good reliability and, < 0.40 considered poor reliability. The standard error of measurement (SEM) presented in the units of the scale (HU) was calculated to estimate the precision of each measurement to give an indication of test to test variability in cortical and trabecular densitometry. Bland-Altman 95% limits of agreement were calculated based on a t-distribution with 9° of freedom due to the small sample size to determine the mean difference and level of agreement between the two test sessions.

## Results

Mean density for each bone for both scan one and two, ICC (95% CI) between scans, limits of agreement and SEM are included in Table 2. Limits of agreement graphs are provided in additional files 1, 2, 3 and 4. Trabecular measurements displayed excellent reliability with ICC’s ranging from 0.81 to 0.91, except for the navicular (0.70), cuboid (0.68) and fourth metatarsal (0.69) which displayed fair to good reliability. Cortical measurements at the talus (0.52), calcaneus (0.59), navicular (0.70), cuboid (0.69), intermediate cuneiform (0.46) and first metatarsal (0.61) displayed fair to good reliability, with the remaining bones displaying poor reliability. Measurement precision as measured by SEM was poorer in less reliable measures, ranging from 2 to 12% of the mean of the HU measurement for the trabecular measurements. The cortical bone SEM ranged from 5 to 19% indicating poorer precision than for the trabecular bone.

**Table 2:** Intra-class correlation coefficients (ICC), 95% confidence intervals (CI), means Bland-Altman (B-A) limits of agreement and standard error of measurement (SEM) for cortical and trabecular densitometry of 12 ft bones (*n* = 10)

	ICC	95% CI	Mean (HU) Session 1	Mean (HU) Session 2	B-A Lower Limit	B-A Upper Limit	SEM
Trabecular							
Talus	0.91^c^	0.67, 0.98	457.88	491.11	−43.46	109.92	10.17
Calcaneus	0.90^c^	0.64, 0.97	231.85	246.37	−50.59	79.63	9.1
Navicular	0.70^b^	0.17, 0.92	367.88	386.1	−123.27	159.70	34.25
Cuboid	0.68^b^	0.47, 0.91	227.12	223.4	−99.76	92.32	24.01
Medial cuneiform	0.83^c^	0.46, 0.96	371.71	378.28	−125.75	138.89	24.12
Intermediate cuneiform	0.88^c^	0.58, 0.97	498.84	493.5	−130.90	120.22	19.23
Lateral cuneiform	0.86^c^	0.54, 0.96	373.96	355.08	−123.68	85.92	17.34
First metatarsal	0.90^c^	0.66, 0.98	248.28	237.65	−82.08	60.83	9.99
Second metatarsal	0.81^c^	0.40, 0.95	309.19	317.91	−137.69	155.14	28.21
Third metatarsal	0.82^c^	0.44, 0.95	280.1	281.72	−100.64	103.87	19.18
Fourth metatarsal	0.69^b^	0.15, 0.91	268.5	271.72	−128.62	135.06	32.45
Fifth metatarsal	0.85^c^	0.50, 0.96	252.35	275.59	−74.44	120.93	16.72
Cortical							
Talus	0.52^b^	−0.12, 0.85	2987.09	3111.51	−762.81	1011.66	271.73
Calcaneus	0.59^b^	−0.21, 0.88	2764.06	2693.04	−775.28	633.26	199.35
Navicular	0.70^b^	0.18, 0.92	2823.12	2779.88	−578.67	492.18	129.64
Cuboid	0.69^b^	0.16, 0.91	2573.46	2862.21	−380.13	957.64	161.96
Medial cuneiform	0.17	−0.48, 0.70	2728.22	2672.93	−1006.28	895.71	382.99
Intermediate cuneiform	0.46^b^	−0.20, 0.83	2699.67	2735.92	−554.57	627.08	191.93
Lateral cuneiform	−0.18^a^	−0.71, 0.47	2668.16	2717.88	−1007.55	1106.99	507.69
First metatarsal	0.61^b^	0.01, 0.89	2897.07	2929.04	−633.15	697.11	183.62
Second metatarsal	−0.17^a^	−0.70, 0.48	2872.36	2894.9	−960.78	1005.87	470.19
Third metatarsal	0.22	−0.44, 0.72	2719.87	2892.68	−674.17	1019.79	330.67
Fourth metatarsal	−0.03^a^	−0.62, 0.58	2728.13	2791.24	−974.57	1100.79	525.02
Fifth metatarsal	0.37	−0.30, 0.80	2704.72	2716.83	−763.82	788.04	272.25

*HU* Hounsfield units

^a^ the ICC obtained was negative due to greater intra-group variation than between group variation in that bone

The measurement is unreliable ^b^fair to good reliability, ^c^excellent reliability

## Discussion

In this study, the reliability of a novel method of assessing foot bone density was determined. The study found excellent reliability of the method in the trabecular bone of most tarsals and metatarsals. Cortical bone displayed fair to good reliability in the talus, calcaneus, navicular, cuboid, intermediate cuneiform and first metatarsal, though was generally poorer than that of trabecular bone.

Precision of three-dimensional analyses of foot bones previously has shown very little error. Commean et al. 2009 [8] examined the precision of three-dimensional whole BMD of the tarsals and metatarsals after segmentation and obtained coefficients of variance ranging from 0.2% for the talus to 1.6% for the fifth metatarsal. Additionally, repeatability of pQCT BMD measurement has been found to be excellent in the second metatarsal in cadavers obtaining an ICC of 0.98 for both cortical and trabecular BMD (mg.cm^3^) [12]. To the author’s knowledge this study is the first to assess reliability of foot bone measurements from multiple CT slices obtained from full foot scans in vivo.

This study found the trabecular measurements to be more reliable than cortical estimates as assessed by both ICC and the Bland-Altman method with cortical measurements having wide 95% confidence intervals and limits of agreement. There was also a lower mean bias between session 1 and 2 in the trabecular measurements. This is probably due the inability to sample the whole cortical bone on the slices in the methodology, resulting in the use of smaller sample regions of interest. It is possible that sampling more regions of interest (more than the nine used in this study) may yield more reliable cortical BMD estimates. The separation of cortical and trabecular bone measures is useful as the two are metabolically distinct and may be affected differently by disease processes [11]. All of the bones were found to have acceptable reliability in the trabecular bone, which is thought to be more susceptible to change during disease processes due to its higher turnover [8]. However, cortical density has been found to be more indicative of fracture risk [14]. Though generally reliability of the cortical bones was lower, fair to good reliability for cortical bone measurement in the talus, calcaneus, navicular, cuboid, intermediate cuneiform and first metatarsal. In these six bones, therefore, this novel method, further developed, could be used to assess the relative effect of disease processes and treatments on these two compartments of bone.

In this study, BMD was not converted to mg of hydroxyapatite (mg.cm^3^), but rather retained the values in HU. Hounsfield units are quantitative units of the radiodensity of objects as obtained from CT scanning where water is calibrated to zero [11]. Hounsfield units are relatively simple to attain and have been associated with bone strength and fracture risk, making them a useful measure [15]. The disadvantage of conversion to mg.cm^3^ is that it requires phantoms that are not readily available in the range of bone densities encountered in the foot [8]. In the interest of developing a practical method of examining foot bone density in the presence of disease, values were therefore left as HU. Now that the reliability of this method has been assessed, such further development of the technique with phantoms is warranted.

Furthermore, in an effort to develop an efficient method to assess fracture risk in the periphery, average densities (HU) were assessed across multiple CT slices rather than perform a time consuming registration and segmentation process. This assessment of the accuracy of this densitometry method is limited to comparison with values in existing literature in a similar population. Commean et al. [8] obtained combined cortical and trabecular BMD (HU) for all tarsals and metatarsals in those with diabetes, peripheral neuropathy and history of ulceration. For example the average density at the calcaneus was 333 HU, the navicular was 481 HU and the first metatarsal was 427 HU. The trabecular values found in this study were almost universally lower than those found by Commean et al. 2009 [8], whilst the cortical values found in this study were considerably higher. However, since Commean et al. 2009 [8] reported the density of the bone inclusive of both trabecular and cortical bone, the current values may be consistent with theirs when considering the relative contribution of trabecular and cortical bone to the overall volume of each bone. It should be noted in the current study, like Commean et al. 2009 [8], this study found significant variation in BMD among the bones of the foot. We therefore recommend that more than one bone is used in assessment, as one foot bone is unlikely to be representative of all foot bones. In particular, we would recommend measurement of those bones that were shown to have fair to good reliability for bone cortical and trabecular bone, i.e., the talus, intermediate cuneiform, calcaneus, cuboid, navicular and first metatarsal.

Several limitations to this study are acknowledged. Repeat scans were performed on the same day meaning that the results represent only same day reliability, although scans were read on different days. Further research should address validity concerns, for example, Smith et al. [16] examined the effect of varying technical and biological parameters on foot BMD estimates from CT to find potential sources of variation. They showed that the impact of simulated soft tissue also resulted in a small amount of variation with an inverse relationship between the amount of soft tissue and resulting HU. Further research should also aim to replicate these results with other CT scanners and operators and in other populations, the 3, 1 model used in the ICC analysis means the results cannot be generalised to other raters. The sample in this study had type 2 diabetes and was largely male and elderly and therefore cannot be generalised beyond this. Finally, due to ethical concerns of duplicate scanning using ionising radiation the sample size was small, possibly the ICC values may have been closer to that found in reliability investigations of previous studies if the sample size was greater.

## Conclusions

This study demonstrated that foot bone density can be reliably measured in those with diabetes by assessing averaged densities from slices of full foot CT scans. These findings offer a relatively simple, quick and reliable method of quantifying foot bone density. This warrants further development to be used as an indicator of risk of foot disease and its progression, to predict treatment outcomes, assess treatment effectiveness and investigate underlying causes of disease states.

## Additional files

Additional file 1: Limits of agreement graphs for cortical bone of a) talus, b) calcaneus, c) navicular, d) cuboid, e) medial cuneiform, f) intermediate cuneiform. (JPG 145 kb)
Additional file 2: Limits of agreement graphs for cortical bone of a) lateral cuneiform, b) first metatarsal, c) second metatarsal, d) third metatarsal, e) fourth metatarsal and f) fifth metatarsal. (JPG 25 kb)
Additional file 3: Limits of agreement graphs for trabecular bone of a) talus, b) calcaneus, c) navicular, d) cuboid, e) medial cuneiform, f) intermediate cuneiform. (JPG 131 kb)
Additional file 4: Limits of agreement graphs for trabecular bone of a) lateral cuneiform, b) first metatarsal, c) second metatarsal, d) third metatarsal, e) fourth metatarsal and f) fifth metatarsal. (JPG 136 kb)

## Abbreviations

BMD: Bone mineral density
CT: Computed tomography
HU: Hounsfield units
ICC: Intra-class correlation coefficient
pQCT: peripheral quantitative computed tomography
SEM: Standard error of measurement
