# Supplementary figures and images for: Computed tomography derived bone density measurement in the diabetic foot
Source: J Foot Ankle Res. 2017 Mar 3;10:11. doi: 10.1186/s13047-017-0192-7 (PMC5335776; doi:10.1186/s13047-017-0192-7)

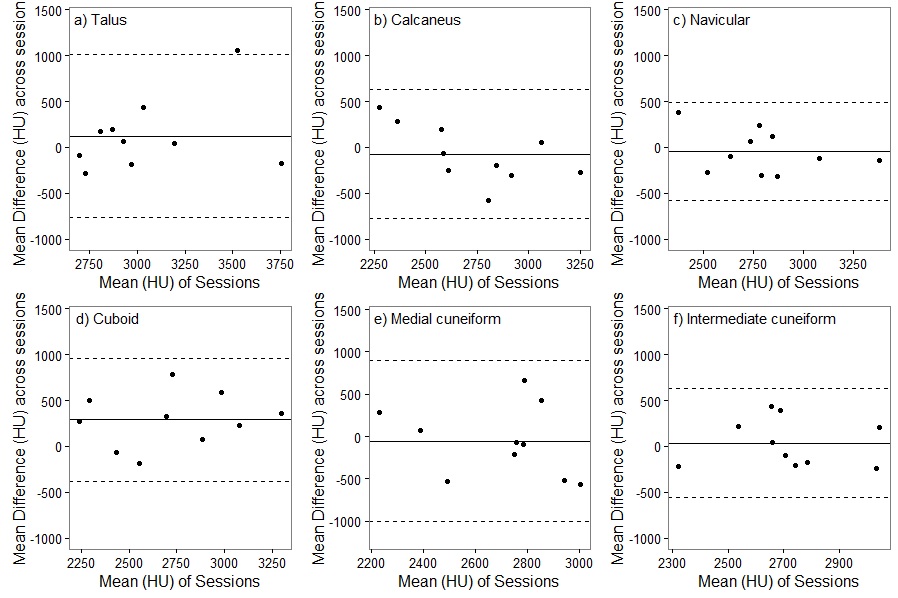

Supplement: Additional file 1: — Limits of agreement graphs for cortical bone of a) talus, b) calcaneus, c) navicular, d) cuboid, e) medial cuneiform, f) intermediate cuneiform. (JPG 145 kb) [file 13047_2017_192_MOESM1_ESM.jpg]

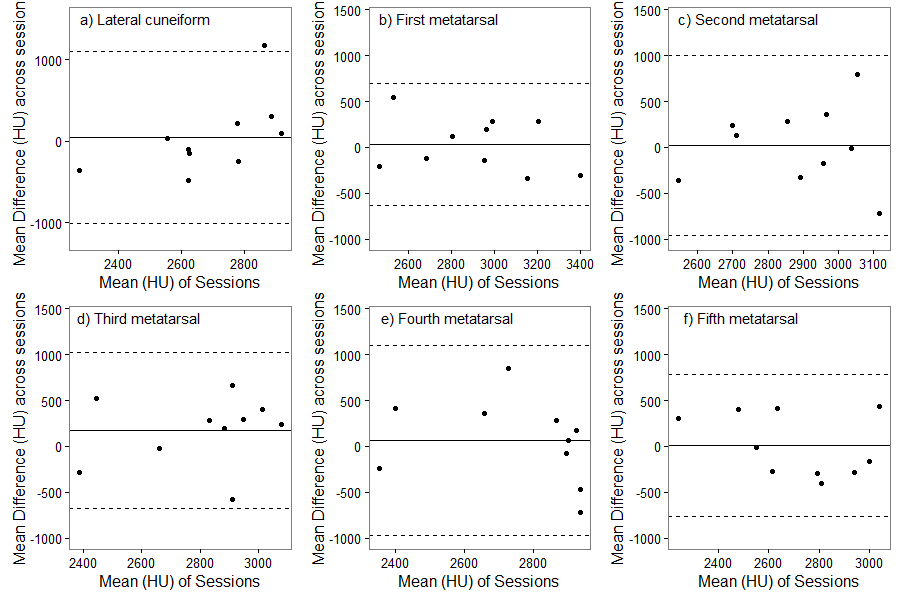

Supplement: Additional file 2: — Limits of agreement graphs for cortical bone of a) lateral cuneiform, b) first metatarsal, c) second metatarsal, d) third metatarsal, e) fourth metatarsal and f) fifth metatarsal. (JPG 25 kb) [file 13047_2017_192_MOESM2_ESM.jpg]

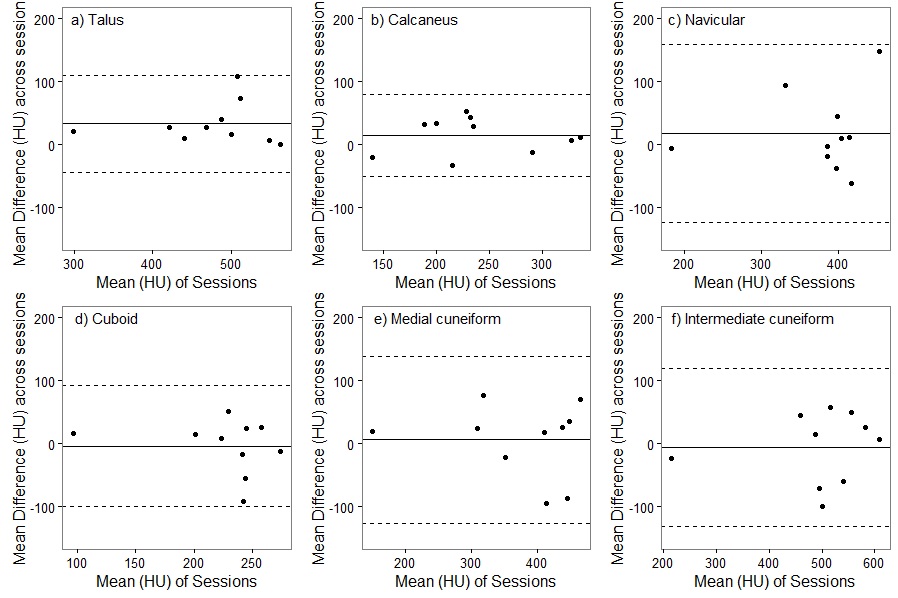

Supplement: Additional file 3: — Limits of agreement graphs for trabecular bone of a) talus, b) calcaneus, c) navicular, d) cuboid, e) medial cuneiform, f) intermediate cuneiform. (JPG 131 kb) [file 13047_2017_192_MOESM3_ESM.jpg]

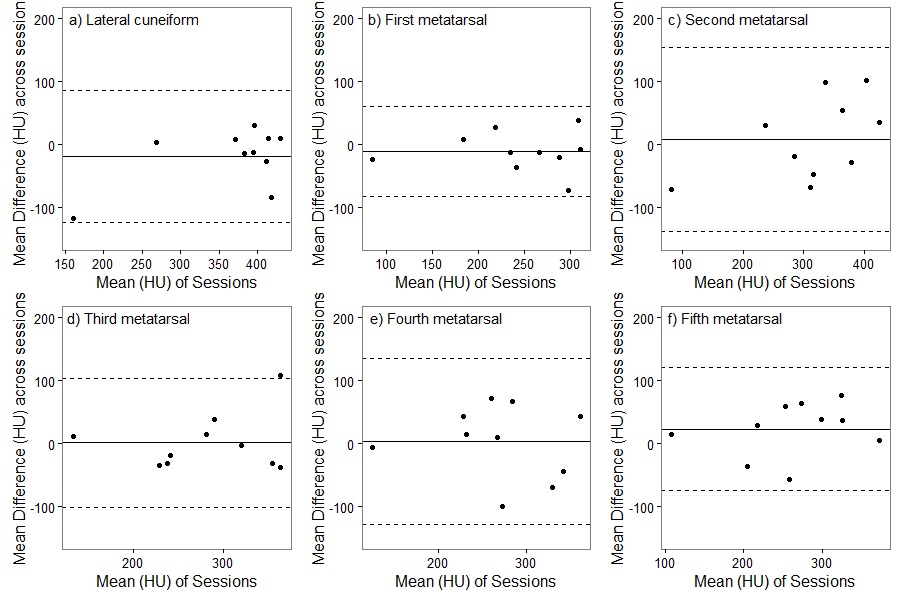

Supplement: Additional file 4: — Limits of agreement graphs for trabecular bone of a) lateral cuneiform, b) first metatarsal, c) second metatarsal, d) third metatarsal, e) fourth metatarsal and f) fifth metatarsal. (JPG 136 kb) [file 13047_2017_192_MOESM4_ESM.jpg]
